# Head-to-head comparison of influenza vaccines in children: a systematic review and meta-analysis

**DOI:** 10.1186/s12967-024-05676-9

**Published:** 2024-10-04

**Authors:** Réka Garai, Ágoston Jánosi, Péter Krivácsy, Vivien Herczeg, Tamás Kói, Rita Nagy, Marcell Imrei, Andrea Párniczky, Miklós Garami, Péter Hegyi, Attila József Szabó

**Affiliations:** 1 Pediatric Center, MTA Center of Excellence, Semmelweis University, Bókay Unit, Bókay János Utca 53-54, 1083 Budapest, Hungary; 2 Centre for Translational Medicine, Semmelweis University, Budapest, Hungary; 3 Heim Pál National Pediatric Institute, Budapest, Hungary; 4 Department of Stochastics, Institute of Mathematics, Budapest University of Technology and Economics, Budapest, Hungary; 5 Institute for Translational Medicine, Medical School, University of Pécs, Pécs, Hungary; 6 Pediatric Center, MTA Center of Excellence, Semmelweis University, Tűzoltó Unit, Budapest, Hungary; 7 Institute of Pancreatic Diseases, Semmelweis University, Budapest, Hungary; 8 ELKH-SE Pediatrics and Nephrology Research Group, Budapest, Hungary

**Keywords:** Flu, Vaccine efficacy, Vaccine safety, Cost-effectiveness, Pain-free vaccines, Health policy

## Abstract

Although vaccination is considered the most effective weapon against influenza, coverage rates, national vaccination policies, and funding vary largely around the globe. Despite their huge potential for achieving herd immunity, child-focused national vaccination strategies that favor pain-free nasal vaccines are uncommon. CENTRAL, Embase, and MEDLINE were last searched on November 13, 2023. Active-controlled randomized controlled trials comparing the live-attenuated intranasal vaccine with the inactivated intramuscular influenza vaccine in children were included. Event rates of laboratory-confirmed influenza virus infection, all-cause mortality, hospitalization, serious adverse events, adverse events, and financial outcomes were extracted based on the PRISMA 2020 Guideline. PROSPERO: CRD42021285412. Pooled odds ratios (ORs) with 95% confidence intervals (CI) were calculated using the random-effects model when at least three comparable outcomes were available. We found no significant difference between quadrivalent live-attenuated intranasal and trivalent inactivated intramuscular (OR = 1.48; 95% CI 0.49–4.45) or between trivalent live-attenuated intranasal and inactivated intramuscular vaccines (OR = 0.77, CI = 0.44–1.34) regarding their efficacy. However, the subgroup analysis of large, multi-center trials indicated that the trivalent live attenuated intranasal influenza vaccine was superior to the trivalent inactivated intramuscular influenza vaccine (12,154 people, OR = 0.50, CI = 0.28–0.88). Only 23 “vaccine-related serious adverse events” were recorded among 17 833 individuals, with no significant difference between methods. The widespread initiation of pediatric national flu vaccination programs prioritizing the live-attenuated intranasal influenza vaccine would be beneficial. Multi-continent, high-quality studies that include children younger than two years old and those living in subtropical and tropical regions are needed to further enhance our understanding.

**Supplementary Information:**

The online version contains supplementary material available at 10.1186/s12967-024-05676-9.

## Background

Globally, approximately 290,000–650,000 deaths occur due to influenza each year [1]. As children are particularly at risk for influenza-related complications, vaccination is recommended for those older than six months, with updated recommendations provided annually [2]. Unfortunately, in most countries, vaccination coverage is not well reported and remains well below the WHO target of 75% [3–6].

Trivalent inactivated intramuscular influenza vaccines (IIV) have been licensed for children since 2001 [7]. They are available for children at least 6 months old, with the only contraindication being a previous anaphylactic or severe allergic reaction [8]. Live attenuated vaccines (LAIV) became available later, in 2007 in the USA and 2011 in Europe, for children older than two years, with several contraindications, such as immunosuppression or aspirin use [8–10]. After the introduction of trivalent vaccines containing two influenza A and one influenza B subtypes, the first quadrivalent vaccines (containing an additional B subtype) were approved in 2012 by the US Food and Drug Administration (FDA) and in 2013 by the European Medicines Agency (EMA) [9–11]. After the “era” of quadrivalent LAIV and IIV, the World Health Organization (WHO), the FDA, and the EMA have recommended reverting to trivalent vaccines starting from the 2024–2025 season [12–14]. Although LAIV might be more accepted by children and families due to its pain-free, potentially self-administrable form, its worldwide use varies, ranging from government-funded school-based vaccination programs to unavailability [15–22].

Meta-analyses on the topic predominantly contain data from observational studies [23, 24]. A recent meta-analysis of test-negative studies suggests similar vaccine effectiveness for all vaccine types, but it included only one study describing trivalent LAIV. Interestingly, it found lower vaccine effectiveness in Asia than in North America, but it could not differentiate between children and adults [24]. According to another meta-analysis, all trivalent vaccines were significantly more effective than placebo, with trivalent LAIV showing superior efficacy [23]. In contrast, no significant difference was observed, when comparing quadrivalent influenza vaccines to a placebo [23]. This study neither included quadrivalent head-to-head randomized controlled trials (RCTs) nor detailed safety or cost information [23].

Thus, we decided to perform a systematic review and meta-analysis, including only head-to-head RCTs, to directly compare the efficacy, safety, and cost-effectiveness of LAIV and IIV in children. If the nasal vaccine proves to be as efficacious, safe, and cost-effective as the intramuscular one, it should form the basis of potential national vaccination strategies to support the WHO’s Global Influenza Strategy 2019–2030 [22, 25].

## Methods

### Selection, data extraction, and quality assessment

We conducted a systematic literature search in MEDLINE (via PubMed), Embase and the Cochrane Register of Controlled Trials (CENTRAL) following the Preferred Reporting Items for Systematic Reviews and Meta-analyses (PRISMA) guidelines [26]. The search was last updated on November 13, 2023, using the following search key: (vaccine OR vaccin* OR immunization OR shot OR injection) AND (influenza OR H1N1 OR H3N2 OR flu OR A/H1N1 OR A/H3N2) AND (pediatric OR paediatric OR child*) AND (random*).

We included randomized, active-controlled trials that investigated children after influenza vaccination, comparing LAIV to IIV if they reported rates of laboratory-confirmed influenza, safety events, or numerical data on cost-effectiveness, with no further restrictions. We also performed a manual search for cited and citing papers (via Google Scholar) of the included studies and relevant reviews. Two independent review authors (RG and ÁJ) performed the selection on Rayyan.ai (Rayyan Systems Inc., 2020) [27]. After duplicate removal, articles were screened based on title, abstract, and full text. Cohen’s Kappa coefficient was calculated after each stage to measure inter-rater agreement. Any conflicts were resolved through discussion and consensus. The following data were extracted by RG and VH when at least three comparable outcomes were available: first author, year of publication, age and health status of the study population, study period and site, treatment type and dose, event rates of laboratory-confirmed influenza after vaccination, reactogenicity, and adverse events.

We deviated from our protocol (PROSPERO CRD42021285412) by including data on individuals up to 21 years of age, allowing us to include two additional relevant articles [28, 29]. Also, we decided to exclude data on mono- or bivalent vaccines, as they are now clinically irrelevant. Due to insufficient data, we could not report on influenza-like illness rates. Additionally, we chose not to analyze data on antibody responses, as the outcome measures were diverse and merely predictive of real-world performance [30]. Finally, we included data from clinical trial registry sites.

### Data analysis

Results are presented as pooled odds ratios (OR) with p-values, 95% confidence intervals (CI), and prediction intervals using the random effects variant of the Mantel–Haenszel method [31, 32]. In the presence of zero frequency, ORs within the studies were calculated by adding 0.5 to the cell frequencies; however, to calculate the pooled ORs, the exact Mantel–Haenszel method was applied to handle zero frequencies without the mentioned correction. To estimate ${\tau }^{2},$ we used the Paule-Mandel method [33] with Hartung-Knapp adjustment [34]. Besides the prediction interval, heterogeneity was assessed by calculating the I2 measure and its CI and performing the Cochrane’s Q test. The results of the meta-analyses are displayed in forest plots. Publication bias was examined via funnel plot in the presence of eight studies and Egger's test when at least ten studies were available. We assessed the influence of individual studies on the overall pooled effect by leave-one-out analysis for the efficacy analysis. The analysis was executed with package `meta` of the R statistical software (version 4.1.2.) in line with Harrer et al. [35].

In the case of overlapping data [36–39], we prioritized those from the peer-reviewed articles. Data for overall serious adverse and adverse events analyses were derived from cumulatively published data. The risk-of-bias tool recommended by the Cochrane Collaboration for randomized trials (RoB2) [40] and the GRADE approach [41] were applied to assess evidence quality by RG and VH.

### Subgroups

Major subgroups were based on the valency of vaccines. We created additional subgroups based on study size, as the detectable number of influenza virus infections is highly determined by its incidence (3–11% in the USA) [42], to decrease the number of zero events and increase generalizability. Additionally, studying large, multi-center studies helped us analyze cumulative data from different geographical sites. We also aimed to investigate the hypothesis that pre-existing immunity in the host can disturb the immunogenicity of LAIV, especially if the contained strain is antigenically similar to a previously acquired one, limiting its nasal replication. This, in combination with children’s developing immune system, could lead to an improved efficacy for seronegative, usually younger children [43, 44]. Therefore, we designed age categories according to age ranges.

## Results

**Fig. 1:**
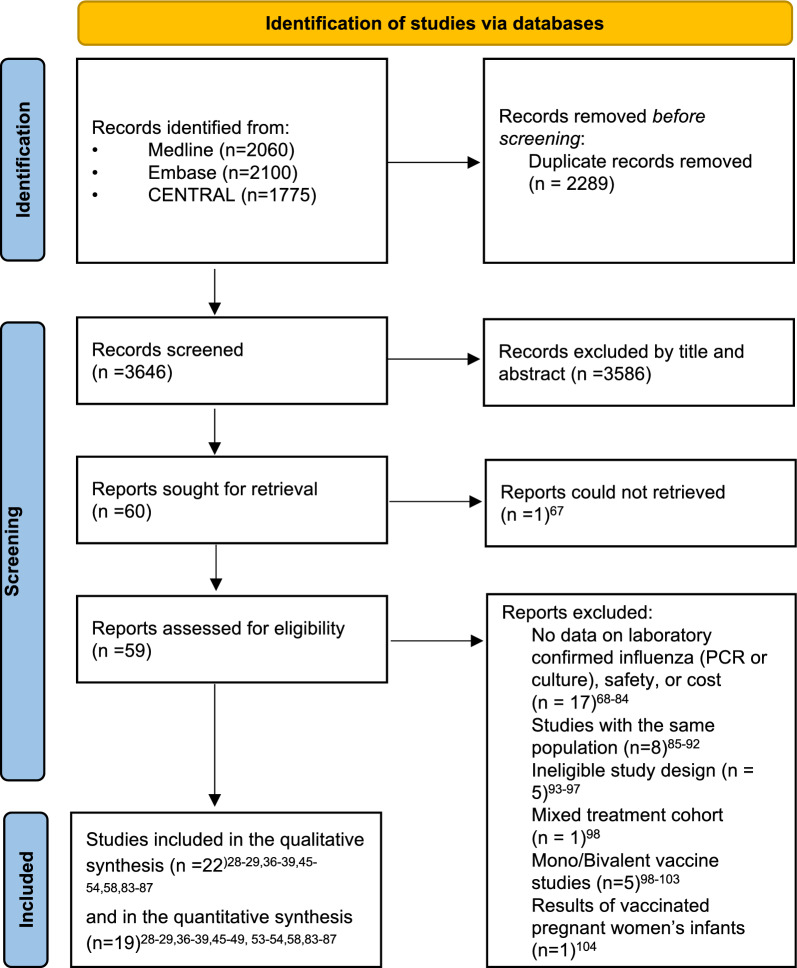
PRISMA 2020 flowchart representing the study selection process

**Fig. 2:**
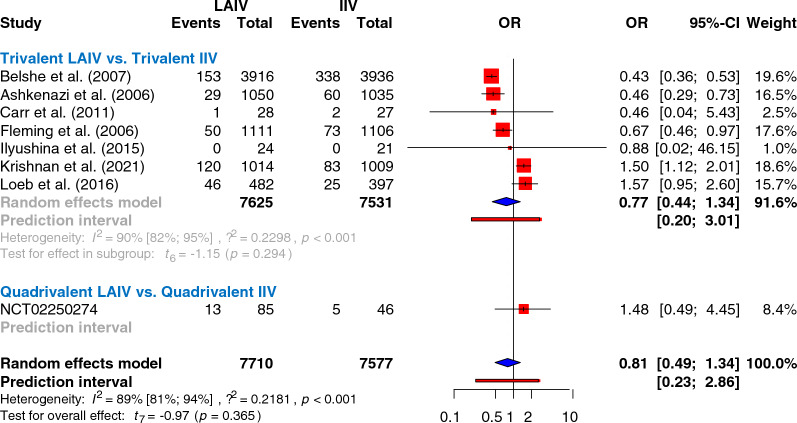
Comparing the incidence of confirmed influenza cases after influenza vaccination presented in odds ratios. (Results of trivalent versus trivalent analyses are separated from the single quadrivalent LAIV versus trivalent IIV trial, presented on the same forest plot)

Of the 3,648 relevant trials, 19 were included in the quantitative synthesis (Fig. 1, Table 1). Studies in the meta-analysis were conducted between 1985 and 2021, including data on 15 156 individuals for the efficacy comparison of trivalent vaccines (Table 1, Fig. 2).

**Table 1:** Basic characteristics

Author (year)	Style	Number of centers	Study period	Mean age (range), years	Health status	Treatments (dose)	Confirmed influenza (Number of children/ event number)	Safety outcomes
Ashkenazi et al. (2006) [58]	Individually-randomized, open-label	> 100 centers	2002–2003	3.0 (0.5–5.9)	Recurrent respiratory tract infections	TLAIV (2) TIV (2)	1050/29 1035/60	Related and overall serious adverse events, adverse events, hospitalization, subfebrility, fever, sore throat, cough, runny nose, otitis media, irritability, headache, decreased appetite, decreased activity, vomiting, muscle- or body ache, chills
Belshe et al. (2007) [37]	Individually-randomized, double -blind	> 100 centers	2004–2005	(0.5–4.9)	Wheezer 6%	TLAIV (1-2^a^) TIV (1-2^a^)	3916/153 3936/338	Related and overall adverse events, hospitalization, subfebrility, wheezing, runny nose
Carr et al. (2011) [83]	cluster randomized, open-label	< 100 centers	2008–2009	10.4 (2.1–21.0)	Cancer	TLAIV (1-2^b^) TIV (2)	28/1 27/2	Related and overall serious adverse events, subfebrility, sore throat, cough, runny nose, irritability, headache, decreased activity, vomiting, muscle- or body ache, chills
Fleming et al. (2006) [46]	Individually-randomized, open-label	> 100 centers	2002–2003	11.0 (6–17.9)	Asthma	TLAIV (1) TIV (1)	1111/50 1106/73	Related serious adverse events, adverse events, hospitalization, subfebrility, fever, sore throat, cough, runny nose, otitis media, irritability, headache, decreassed appetite, decreased activity, vomiting, upper respiratory tract infections, nasopharyngitis, muscle- or body ache, chills, asthma attack
Hoft et al. (2011) [29]	Individually randomized, open-label	< 100 centers	2005–2006	(0.5–2.9)	Healthy	TLAIV (2) TIV (2)	Not included	Subfebrility, fever, runny nose
		< 100 centers	2006–2007	(1.0–2.9)				
Ilyushina et al. (2015) [38]	Individually randomized, open-label	< 100 centers	2010–2012	5.4 (2.25–9.7)	Healthy	TLAIV (1–2) TIV (1–2)	13/0 18/0	Subfebrility, fever, upper respiratory tract infections
Krishnan et al. (2021) [53]	Individually-randomized, triple -blind	< 100 centers	2015–2017	(2.0–10.0)	Half of children is malnourished	TLAIV (1) TIV (1-2^b^)	1087/279 1092/287	Related and overall serious adverse events, subfebrility, irritability, headache, decreased appetite, decreased activity, vomiting, diarrhea
Kwong et al. (2015) [84]	Cluster randomized, open-label	< 100 centers	2013–2014	(4.0–14.0)	Children	TLAIV (1-2^b^) TIV (1-2^b^)	Not included	Adverse events
Levin et al. (2008) [85]	Individually randomized, open-label	< 100 centers	2004–2005	(5.0–17.9)	HIV	TLAIV (1) TIV (1)	Not included	Skin reactions, nasopharyngitis, ear and eye reactions
Loeb et al. (2016) [54]	Cluster randomized, double-blind	< 100 centers	2012–2015	(3.0–15.0)	Healthy	TLAIV (1-2^b^) TIV (1-2^b^)	1473/91 1201/77	Related serious adverse events, adverse events, hospitalization, subfebrility, sore throat, skin reactions, runny nose, headache, decreased appetite, vomiting, muscle- or body ache, ear and eye reactions, diarrhea, chills
Luce et al. (2021) [50]	Cost-effectiveness modeling study of data of the 2.0–4.9 years old cohort from the Belshe et al. (2007) [37] study							
Neuzil et al. (2001) [86]	Individually randomized, double -blind	< 100 centers	1985–1990	(1.0–16.0)	Healthy	TLAIV (1) TIV (1)	Not included	Related and overall serious adverse events, subfebrility, sore throat, cough, runny nose
Smolen et al. (2014) [52]	Cost-effectiveness modeling study of data from the Ashkenazi et al. (2006) [58] and Fleming et al. (2006) [46] studies							
Sokolow et al. (2022) [49]	Cluster randomized, open-label	< 100 centers	2018–2020	(5.0–17.0)	Asthma	QLAIV (1) QTIV (1)	Not included	Runny nose, sore throat, chills, ear and eye reactions, headache, muscle-or body ache, decreased activity, skin reactions, fever, wheezing, asthma attack, cough
Tarride et al. (2012) [51]	Cost-effectiveness modeling study of three age cohorts of Canadian children: 2.0–5.0 years of age, 6.0–9.0 years of age, and 10.0–17.0 years of age. Vaccine efficacy was taken from the Belshe et al. (2007) [37] study							
EU-CTR 2004–000585-13 [36]	Individually-randomized, double -blind	> 100 centers	2004–2005	(0.5–4.9)	Healthy	TLAIV (1-2^a^) TIV (1-2^a^)	Not included	Mortality, people affected by related serious adverse events, adverse events, skin reactions, otitis media, upper respiratory tract infections, nasopharyngitis, ear and eye reactions, diarrhea, asthma attack
NCT00461981 [87]	Individually randomized, open-label	< 100 centers	2007–2008	(1.0–2.9)	Healthy	TLAIV (2) TIV (2)	Not included	Related serious adverse events, adverse events, subfebrility, cough, runny nose, vomiting, upper respiratory tract infections, nasopharyngitis, diarrhoea
NCT01194297 [48]	Individually randomized, open-label	< 100 centers	2010–2011	(2.0–3.0)	Premature, very low birth weight and former full-term infants	QLAIV (1) QIV (1)	Not included	Related and overall serious adverse events, adverse events
NCT01246999 [39]	Individually randomized, open-label	< 100 centers	2010–2011	(3.0–9.0)	Healthy	TLAIV (2) TIV (2)	Not included	Related and overall serious adverse events, subfebrility, sore throat, wheezing, cough, runny nose, headache, decreased activity, muscle- or body ache
NCT02250274 [45]	Individually randomized, open-label	< 100 centers	2014–2015	10.0 vs.12.0^d^ (5.0–17.0)	Healthy	QLAIV (1) TIV (1)	85/13 46/5	Related and overall serious adverse events
NCT03600428 [47]	Individually randomized, open-label	< 100 centers	2018–2020	(5.0–11.0)	Asthma	QLAIV (1) QIV (1)	Not included	Mortality, related and overall serious adverse events, wheezing, cough, asthma attack
NCT03982069 [28]	Individually randomized, open-label	< 100 centers	2019–2021	(4.0–21.0)	Healthy	QLAIV (1) QIV (1)	Not included	Mortality, related and overall serious adverse events, adverse events

TLAIV: trivalent live attenuated influenza vaccine; TIV: trivalent inactivated influenza vaccine; QLAIV: quadrivalent live attenuated influenza vaccine; QIV: quadrivalent inactivated influenza vaccine

^a^Second dose, if none before

^b^Second dose, if < 9 years old, less than 2 prior vaccinations

^c^Second dose, if post-vaccination serum HAI titers against H1N1 and H3N2 ≤ 8 were revaccinated

^d^Mean age was 10 in the LAIV group, 12 in the TIV

### Efficacy

The only active-controlled RCT incorporating results on confirmed influenza virus infection rates was a Phase IV clinical trial conducted in 131 children aged 5–17 years, which resulted in no significant difference between quadrivalent LAIV and trivalent IIV [45] (Fig. 2).

**Fig. 3:**
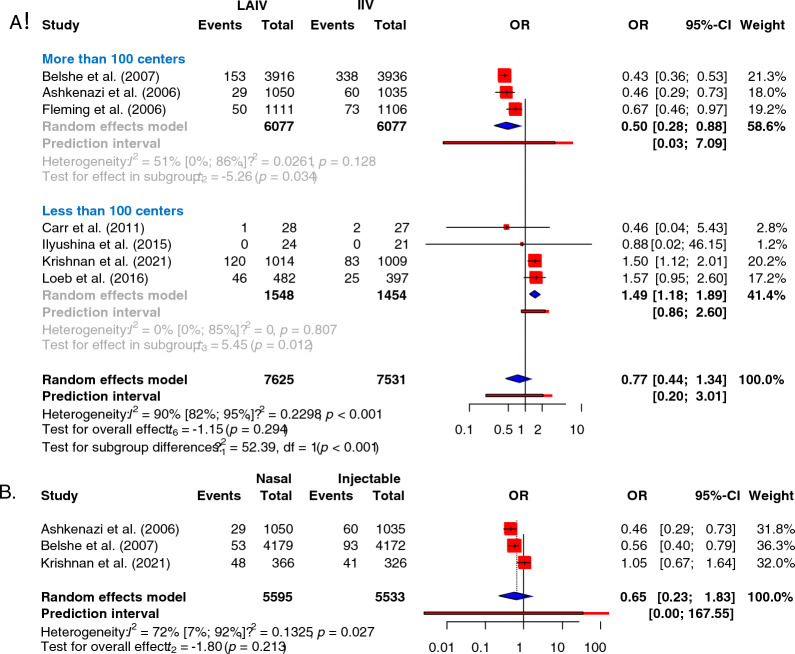
Subgroup analyses of the trivalent efficacy analysis.** A** Comparing larger, multi-center studies with smaller studies. Large, multi-center studies (more than a 100 centers), smaller studies (less than a 100 centers). **B** Results of children younger than 6 years of age

When comparing trivalent vaccines directly, there was no significant difference between the methods in the odds of reducing flu infections after vaccination (Fig. 2). No study was identified as significant by the leave-one-out sensitivity analysis (Supplementary Fig. 1). Although we found LAIV superior in large, multi-center studies, we observed the opposite result in smaller trials (Fig. 3A). There was no significant difference between trivalent vaccines when analyzing data from children younger than six years (Fig. 3B).

### Safety

Data from 8958 children obtained from clinical trial protocol sites showed no vaccination-related deaths (Supplementary Fig. 2A).

The odds of experiencing serious adverse events (SAEs) were significantly higher in the trivalent nasal vaccine group than in the trivalent injectable group (Table 2, Supplementary Fig. 2Ba). No reported SAEs were found regarding quadrivalent vaccines (Supplementary Fig. 2Ba–c). We observed no significant difference between the groups when investigating the odds of SAEs based on the number of affected individuals (Table 2, Supplementary Fig. 2Bb). Only 23 “vaccine-related SAEs” were recorded among 17,833 individuals (none after quadrivalent vaccines), with no significant difference between trivalent or quadrivalent methods (Table 2, Supplementary Fig. 2Bc); even when investigating children younger than nine years (Table 2, Supplementary Fig. 2Bd).

**Table 2:** Pooled safety results of trivalent vaccines displayed in sequence of the level of GRADE and effect size

Lower odds for the nasal group			Lower odds for the intramuscular group			
Outcome	OR (CI 95%)	GRADE	Outcome	OR (CI 95%)	GRADE	
			Nasal symptoms—large, multi-center studies	1.64 (1.33; 2.02)	High	Significant
			Nasal symptoms	1.55 (1.30; 1.86)	Moderate	
			Adverse events by the number of affected people	1.26 (1.14;1.40)	Moderate	
			Serious adverse events	1.17 (1.02;1.34)	Moderate	
Coughing	0.95 (0.86;1.05)	High	Significant wheezing	1.16 (0.86; 1.56)	High	Non-significant
Diarrhea	0.98 (0.83; 1.16)	High	Sore throat	1.14 (0.99; 1.32)	High	
Related serious adverse events under nine years of age	0.93 (0.53; 1.63)	Moderate	Vomiting	1.09 (0.78; 1.53)	Moderate	
Chills	0.74 (0.26; 2.11)	Low	Serious adverse events by number of affected people	1.11 (0.96;1.28)	Low	
Having a temperature higher than 38,5C	0.78 (0.37; 1.66)	Low	Decreased activity	1.02 (0.59; 1.76)	Low	
Headache	0.90 (0.62;1.31)	Low	Otitis media	1.48 (0.45; 4.89)	Very low	
Adverse events by the number of reported events	0.51 (0.05;5.09)	Very low	Upper respiratory tract infection	1.64 (0.27; 10.05)	Very low	
Muscle- or body ache	0.56 (0.24; 1.32)	Very low	At least subfebrility under six years of age	1.32 (0.46; 3.75)	Very low	
Hospitalization	0.58 (0.04;7.86)	Very low	Skin reactions	1.27 (0.14;11.65)	Very low	
Adverse events	0.81 (0.36;1.83)	Very low	Nasal symptoms—smaller studies	1.24 (0.89; 1.71)	Very low	
Fever	0.85 (0.50;1.43)	Very low	Nasopharyngitis	1.22 (0.53; 2.79)	Very low	
At least subfebrility above six years of age	0.91 (0.58; 1.43)	Very low	Irritability	1.08 (0.78; 1.49)	Very low	
At least subfebrility	0.93 (0.54; 1.61)	Very low	Related serious adverse events	1.07 (0.70;1.62)	Very low	
Decreased appetite	0.94 (0.66; 1.35)	Very low	Ear or eye reactions	1.06 (0.47; 2.40)	Very low	
Wheezing	0.98 (0.73; 1.33)	Very low				

Overall, 316 patients (LAIV:1.29% vs. IIV:1.34%) were hospitalized, with a tendency for fewer hospitalizations after administering trivalent LAIV (Table 2, Supplementary Fig. 2C).

A tendency for fewer adverse events (AEs) was observed after administering the trivalent nasal vaccine when comparing event rates (Table 2, Supplementary Fig. 2D). However, when evaluated by the number of affected individuals, there was a significant difference, with lower odds after the trivalent injectable form (Table 2, Supplementary Fig. 2D).

We compared 21 types of AEs experienced after administering trivalent vaccines (Table 2, Supplementary Fig. 2A–Yb). A significant difference was observed only for “nasal symptoms”, with lower odds after the injectable form (Table 2, Supplementary Fig. 2R). We could not meta-analyze asthma exacerbations, but according to the two available studies, there was no significant difference in the odds ratios (OR = 1.08 95% CI 0.90; 1.29 [46] and OR = 1.12 95% CI 0.78;1.60 [36]) (Supplementary Fig. 2C). Regarding quadrivalent vaccines, only coughing (OR = 0.88 95% CI 0.44;1.76) [47], significant wheezing (OR = 0.62 95% CI 0.31;1.21 [47] and OR = 1.67 95% OR = 0.02;137.35 [48]) and asthma exacerbations (OR = 0.70 95% CI 0.26;1.90) [49] were evaluated, with no significant difference between methods; however a meta-analysis could not be conducted (Supplementary Figs. 2H, Yb, E).

### Cost-effectiveness

Three studies assessed cost-effectiveness based on the results of previous trivalent RCTs (Table 1). We could not meta-analyze the data, as confidence intervals of mean costs were reported by only one study [50]. Nonetheless, all studies concluded that LAIV was financially more beneficial [50–52]. The explanation behind this is that although the administration of the nasal vaccine was more expensive, in the long term, with the reduction of influenza cases, healthcare utilization, and productivity loss of parents, LAIV resulted in direct and societal savings, making it more favorable for a potential child-focused national vaccination strategy [50–52].

We found no study comparing quadrivalent RCTs.

### Quality assessment

The overall risk of bias in the efficacy analyses was low for all but one study, which raised some concerns in the “deviations from the intended interventions” domain, as the number of patients lost to follow-up was high but accounted for. Two other studies raised concerns regarding the “selection of the reported results” domain due to the lack of statistical analysis plan protocols (Supplementary Table 1). The overall bias in the safety analysis was high in 6 out of 19 studies. (Supplementary Table 2).

The certainty of evidence of trivalent LAIV being more efficacious is “high” based on large, multi-center trials. Over half (24/34) of trivalent safety analyses were reported as having a “low” or “very low” grade of evidence (Table 2). All three quadrivalent safety analyses have a “very low” level of evidence. Detailed GRADE results can be seen in Supplementary Table S3, S4.

We performed Egger’s tests for one analysis, and funnel plots for four analyses, with no evidence of publication bias (Supplementary Fig. S3–S6.).

## Discussion

Our analysis of trivalent vaccines, involving data from 15,156 children, found no significant difference between LAIV and IIV in the odds of having a confirmed influenza virus infection after vaccination, indicating similar efficacy. When analyzing data from large, multi-center trials, which are more likely to be generalizable, the nasal vaccine was significantly more efficacious than IIV. In contrast, opposing results were observed in smaller studies. Two trials particularly influenced these outcomes, favoring the application of IIV. One was conducted mainly on malnourished Indian children [53], and the other was based in a Hutterite community [54]. Race and ethnicity-related disparities such as group living conditions, limited access to healthcare, intrinsic variables, and vaccine uptake rates can regulate susceptibility. Additionally, in 2014–2015, when the dominant strain was a poor antigenic match for both vaccines, very low vaccine efficacy [10] may have influenced the results of the Loeb et al. [54] and NCT02250274 [45] studies. Also, in 2015–16, when LAIV was considered less protective against H1N1 strains [10], the dominant isolate was the 2009 H1N1, possibly impacting the Krishnan et al. [53] trial as well.

Although we could not verify the age-related hypothesis of trivalent LAIV performing better in younger children [42], we observed a tendency for lower odds of infections after the nasal vaccine was administered to children younger than six years compared to IIV. Encouragingly, based on a recent test-negative, multi-center European trial conducted at the beginning of the 2022–2023 season, higher influenza vaccine effectiveness was observed in children (50–90%) compared to adults, further supporting the viability of child-focused national vaccination strategies [22, 55]. Unfortunately, as the type and valency of vaccines were mixed, the study did not determine if one vaccine type was superior to others [55].

We did not find any published RCTs directly comparing the rates of confirmed flu infections after quadrivalent nasal versus quadrivalent injectable influenza vaccines in children. Only a small trial from clinicaltrials.gov compared quadrivalent LAIV to trivalent IIV, finding no significant difference [45].

Efficacy can also be described by comparing immunogenicity. However, it is important to note that antibody responses are only predictors of real-world performance. Yet, a meta-analysis from 2020 reported similar outcomes for common influenza strains in both quadrivalent vaccines in children; LAIV had significantly higher immunogenicity for the unique B lineage than IIV, providing broader protection [56], which could be attributed to LAIV’s ability to elicit both humoral and cellular immune responses [44].

The second key factor affecting decision-making is safety. It is important to acknowledge that vaccines are among the safest medical products available [57]. A meta-analysis from 2004 found no evidence that flu vaccination results in any significant risk of developing clinically significant adverse events (1 in every 250,000 cases) [22]. However, the discussion of which method is ‘safer’ to use is important.

When coincidental deaths are temporally associated with vaccination, causality is naturally questioned [57]. Although we could not meta-analyze data on all-cause mortality, we reassuringly did not observe any vaccination-related deaths [28, 36, 47].

Our trivalent analysis showed significantly more SAEs after LAIV. However, it should be noted that the two highest-weighted studies were conducted in children with respiratory diseases [58] and in those younger than 5 years [37]. Additionally, when we analyzed SAEs declared to be related to vaccination, their number was distinctively lower in the above-mentioned groups, indicating no difference between vaccines. Moreover, we found no reported SAEs in terms of quadrivalent vaccines [28, 45, 47, 48]. A recent systematic review investigating the safety of LAIV in people with asthma [59] and a novel RCT comparing quadrivalent vaccines in asthmatic children [49] also concluded that there were no safety concerns.

Our finding for hospitalization rates is similar to those reported by Minozzi et al., with no significant difference between trivalent vaccine types [23]. However, we did not find data concerning quadrivalent vaccines. Since the 2013–2014 season, a universal pediatric flu vaccination program utilizing LAIV has been introduced in the United Kingdom. Their recent test-negative study investigating laboratory-confirmed infections resulting in hospitalization estimated that LAIV provided effective protection over three seasons, highlighting the advantage of such programs. This is further supported by the fact that this study includes the 2015–2016 season, when LAIV’s usage was temporarily suspended in favor of IIVs in the United States due to observed lower effectiveness, which they did not detect [60].

According to our trivalent analysis, the injectable form seemed preferable regarding AEs based on the number of affected individuals, but data on children with respiratory diseases strongly influenced this result as well [46, 58]. For overall AEs there was no significant difference. Minozzi et al. did not report more systemic AEs after any of the vaccines compared with placebo, but they did report significantly more local AEs after administering LAIV [23]. To provide a more detailed picture of safety, we compared 21 types of AEs and found no significant difference for all but one (nasal symptoms), with lower odds after trivalent IIV (dominated by results of children with respiratory diseases [46, 58] and those younger than 5 years [37]). The age restriction on LAIV’s application is partially based on the observed higher rates of wheezing in children younger than 24 months [20, 50]. Our analyses of wheezing showed no significant difference between trivalent vaccines, but we could not investigate different age groups or quadrivalent vaccines. Only two trials reported the rates of coughing, wheezing, and asthma exacerbations after quadrivalent vaccines. Sokolov et al. [49] concluded that there was no increase in the frequency of asthma exacerbations and asthma-related symptoms after quadrivalent LAIV compared with IIV in children older than 5 years with persistent asthma [48].

## Strength and limitations

As regulatory bodies advise conducting active-controlled trials when proven therapies exist, our main objective was to investigate head-to-head RCTs across multiple aspects, providing a comprehensive interpretation to determine which influenza vaccination strategy could best serve our patients, aiming for the highest level of evidence [61–63].

The primary limitation of our study—the low number of included quadrivalent studies – can be attributed to the recent trend of conducting less expensive and ethically less questionable test-negative studies to investigate vaccine effectiveness [64, 65]. These studies can provide valuable information relevant to real-world settings but are also at risk for unmeasured confounding, indication, and collider stratification biases [66, 67]. We should also emphasize the drawbacks of RCTs, which are considered the gold standard for evaluating vaccine efficacy; they are more expensive, time-consuming, and sometimes not feasible or ethical to perform [68]. Additionally, handling the results of active-controlled studies requires careful consideration, as the lack of a placebo group introduces the possibility of detecting similarly low or high effectiveness for both vaccine types [62]. Thus, knowing the efficacy of currently available vaccines against placebo is essential.

Another limitation is the high heterogeneity and risk of bias in several analyses. As immunogenicity is influenced by previous influenza vaccinations and infections, the degree of matching strains, and the receiver’s individual characteristics [69], it should be acknowledged that finding homogeneous studies might be difficult due to the nature of the topic.

Investigating safety outcomes presents multiple challenges [30], as we encountered diverse, overlapping, or missing safety definitions (Supplementary Table S5, S6) [70]. Therefore we did not differentiate between reactogenicity and adverse events. Another interesting issue is the relatedness of different AEs to vaccination in a population already at high risk of experiencing irritability, appetite changes, diarrhea, nasopharyngitis, and other conditions, especially in autumn when immunization occurs [71, 72].

Overall, the rigorous methodology -including the pre-study PROSPERO protocol, RoB2 analysis, GRADE assessment, and adherence to PRISMA 2020 guidelines—along with the thorough implications for policymakers and for future research, enhances the value of our study. This is a great example of how translational medicine can contribute to improved medical care [41, 73, 74].

## Implications for policymakers

It is crucial to identify predictors for immunization, as a survey from 2019 reported that 26.0% of parents were unsure about vaccination [75]. This finding aligns with an Italian study from 2022, which reported that only 29.0% of parents were advised by their doctor to vaccinate their children against flu, and 32.5% of them were unaware that it is recommended for children. Unfortunately, 72.7% chose not to vaccinate even after acknowledgment, although 40.2% said they might consider it if it could be administered without an injection. Additionally, of those choosing to vaccinate, 83.0% preferred the needle-free option [21].

As we live in a globalized, aging world in a pandemic era, alongside stable or even increasing influenza-related mortality over the years, there is a tremendous need to re-evaluate current prevention strategies [22]. A “no one size that fits all” strategy is also under discussion because due to countries’ varying latitudinal spread and seasonality [6]. We also highlight the massive difference in the number of studies conducted in the Northern Hemisphere compared to the Southern [24], which confirms the need for a deeper evaluation of influenza vaccination’s hemispheric recommendations [76, 77].

Achieving herd immunity through national child-focused vaccination programs could decrease the epidemic-related health, social, and financial burden, as it is estimated that with 90% vaccination coverage, the incidence could decrease by two-thirds in children and nearly 80% in the most vulnerable elderly population [22]. Unfortunately, there are many countries where the use of LAIV is neither available nor governmentally funded [15–18]. Countries, such as Hungary, where vaccination programs are highly effective (over 90% vaccination rates; National Insurance covers mandatory vaccines, a health visitor network, and some of the recommended vaccines), should consider establishing a pediatric national influenza vaccination program similar to that of the United Kingdom [22, 60, 78]. As our data supplement the existing literature of LAIV being at least as efficacious and safe for children without contraindications, and given that vaccine hesitancy against recommended vaccinations is estimated to be high (44.8%), the vaccine of choice should be pain-free [19, 78, 79]. The potential to achieve herd immunity lies in the prospects of higher acceptance rates; therefore, introducing a government-funded influenza policy for children is estimated to be cost-effective in the long term [80, 81].

## Implications for research

Before the era of universal influenza vaccines, multi-continent, long-term high-quality studies with yearly revaccination, investigating children with different health conditions and age groups (especially those under 2 years), would be highly beneficial. It is crucial to support the decision-making process of countries in the subtropics and tropics with evidence, as nearly half of the world’s population lives in these regions and might face an even greater burden of influenza [6]. They might benefit even more from LAIV’s higher production yield, lower cost, and mode of application [82]. Guidelines are required for uniform safety definitions.

## Conclusion

Based on randomized controlled trials, live-attenuated intranasal vaccines are at least as efficacious and safe as inactivated intramuscular influenza vaccines for those without contraindications. Additionally, they are presumed to be more cost-effective. Therefore, we recommend the initiation of pain-free pediatric national flu vaccination programs with live-attenuated nasal influenza vaccines, given their potential to achieve the WHO’s influenza strategy goals. There is a decisive need for multi-continent, high-quality influenza studies to improve influenza control, especially for countries in the subtropics and tropics where LAIV’s availability is critically low.

## Supplementary Information

Additional file 1

## Data Availability

The datasets used in this study can be found in the full text articles.

## Abbreviations

CI: Confidence interval
FDA: Food and Drug Administration
IIV: Inactivated intramuscular influenza vaccine
ÁJ: Ágoston Jánosi
LAIV: Live attenuated vaccine
OR: Odds ratio
PRISMA: Preferred Reporting Items for Systematic Reviews and Meta-analyses
RCT: Randomized controlled trial
RG: Réka Garai
RoB2: Risk-of-bias tool recommended by the Cochrane Collaboration for randomized trials
USA: United States of America
VH: Vivien Herczeg
