# Supplementary material for: Head-to-head comparison of influenza vaccines in children: a systematic review and meta-analysis
Source: J Transl Med. 2024 Oct 4;22:903. doi: 10.1186/s12967-024-05676-9 (PMC11453075; doi:10.1186/s12967-024-05676-9)
Supplement: Supplementary file 1 — Additional file 1 [file 12967_2024_5676_MOESM1_ESM.docx]

**Head-to-head comparison of influenza vaccines in children: A systematic review and meta-analysis**

**Supplementary Materials**

**Authors**

Réka Garai, MD^1,2^; Ágoston Jánosi, MD^2,3^; Péter Krivácsy, MD^1,2^; Vivien Herczeg, MD^1^; Rita Nagy, MD^2,3,5^; Marcell Imrei, MD^2,3,^; Tamás Kói, PhD^2,4^; Andrea Párniczky MD, PhD^2,3,5^; Miklós Garami MD, PhD, Dr.Med Habil^1,2^; Péter Hegyi, MD, PhD, DSc, Dr. Med Habil^2,5,6^; Attila József Szabó, MD, PhD, DSc, Dr. Med Habil^1,2,7^

**Affiliations:**

1. Pediatric Center, MTA Center of Excellence, Semmelweis University, Bókay Unit, Budapest, Hungary;
2. Centre for Translational Medicine, Semmelweis University, Budapest, Hungary;
3. Heim Pál National Pediatric Institute, Budapest, Hungary;
4. Department of Stochastics, Institute of Mathematics, Budapest University of Technology and Economics, Budapest, Hungary;
5. Institute for Translational Medicine, Medical School, University of Pécs, Pécs, Hungary;
6. Division of Pancreatic Diseases, Heart and Vascular Center, Semmelweis University, Budapest, Hungary
7. ELKH-SE Pediatrics and Nephrology Research Group

**Corresponding author**

Réka Garai MD,

Postal address: H-1083 Budapest, Bókay János utca 53-54, Hungary

E-mail address: [garai.reka@semmelweis.hu](mailto:garai.reka@semmelweis.hu)

Table of Contents

[Supplementary Table 1. Risk of bias assessment of studies included in the efficacy analysis 4](#_Toc174060488)

[Supplementary Table 2. Risk of bias assessment of studies included in the safety analysis 5](#_Toc174060489)

[Supplementary Table 3. Results of the trivalent GRADE assessment 6](#_Toc174060490)

[Supplementary Table 4. Results of the quadrivalent GRADE assessment 10](#_Toc174060491)

[Supplementary Table 5 . Definitions of serious adverse events reported in the included studies 11](#_Toc174060492)

[Supplementary Table 6. Definitions of adverse- and reactogenicity events reported in the included studies 12](#_Toc174060493)

[Supplementary Figure1. Leave-one-out Sensitivity Analysis regarding the efficacy of influenza vaccines 14](#_Toc174060494)

[Supplementary Figure2. Detailed forest plots of the safety analyses 15](#_Toc174060495)

[We compared the incidence of different safety features after influenza vaccination presented in odds ratios (results of trivalent versus trivalent-, and quadrivalent versus quadrivalent vaccine studies are presented separately) 15](#_Toc174060496)

[A) All-cause mortality 15](#_Toc174060497)

[B) Serious adverse events 15](#_Toc174060498)

[a) Serious adverse events (event number) 15](#_Toc174060499)

[c) Vaccine-related serious adverse events 16](#_Toc174060500)

[This figure summarizes the odds ratios comparing the incidence of those serious adverse events, which’s occurrence can be directly related to influenza vaccination (study conductor’s opinion) after vaccination between IIV and LAIV stratified by subgroups based on vaccine valency (Trivalent or Quadrivalent 16](#_Toc174060501)

[d) Vaccine-related serious adverse events under nine years (trivalent vaccines) 17](#_Toc174060502)

[C) Hospitalization 17](#_Toc174060503)

[D) Adverse events 17](#_Toc174060504)

[This figure summarizes the odds ratios comparing the incidence of adverse events after vaccination between trivalent IIV and LAIV stratified by subgroups based on adverse event reporting (“Based on the number of affected people”: events were reported based on the number of people who experienced adverse events throughout a period of time regardless of the number of episodes; “Based on event number”: event number was reported regardless of the number of reporting people). 17](#_Toc174060505)

[E) Asthma 18](#_Toc174060506)

[F) At least subfebrility 18](#_Toc174060507)

[a) Main analysis 18](#_Toc174060508)

[b) Subgroup analysis based on age (6 years) 18](#_Toc174060509)

[G) Chills 19](#_Toc174060510)

[H) Coughing 19](#_Toc174060511)

[I) Decreased activity 19](#_Toc174060512)

[J) Decreased appetite 20](#_Toc174060513)

[K) Diarrhea 20](#_Toc174060514)

[L) Ear or eye reactions 20](#_Toc174060515)

[M) Fever 20](#_Toc174060516)

[N) Having a temperature higher than 38.5C 21](#_Toc174060517)

[O) Headache 21](#_Toc174060518)

[P) Irritability 21](#_Toc174060519)

[Q) Muscle- or body ache 21](#_Toc174060520)

[R) Nasal symptoms 22](#_Toc174060521)

[S) Nasopharyngitis 22](#_Toc174060522)

[T) Otitis media 22](#_Toc174060523)

[U) Skin reactions 22](#_Toc174060524)

[V) Sore throat 23](#_Toc174060525)

[W) Upper respiratory tract infections 23](#_Toc174060526)

[X) Vomiting 23](#_Toc174060527)

[Y) Wheezing 24](#_Toc174060528)

[a) Main analysis 24](#_Toc174060529)

[b) Significant wheezing 24](#_Toc174060530)

[Publication bias, funnel plots, Egger’s tests 25](#_Toc174060531)

[Supplementary Figure 3. Funnel plot of the nasal symptoms analysis 25](#_Toc174060532)

[Supplementary Figure 4. Funnel plot of the vaccine-related serious adverse events analysis 26](#_Toc174060533)

[Supplementary Figure 5. Funnel plot and Eggers’ test of the “at least subfebrility” analysis 27](#_Toc174060534)

[Supplementary Figure 6. Funnel plot of the “at least subfebrility ” subgroup analysis (younger or older than six years) 28](#_Toc174060535)

# **Supplementary Table 1. Risk of bias assessment of studies included in the efficacy analysis**

| **Study name** | **Randomization process** | **Timing of identification or recruitment of participants** | **Deviations from intended interventions** | **Missing outcome data** | **Measurement of the outcome** | **Selection of the reported result** | **Overall risk of bias** |
| --- | --- | --- | --- | --- | --- | --- | --- |
| Ashkenazi et al. (2006) | 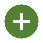 | N.A. | 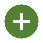 | 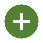 | 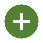 | 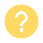 | 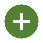 |
| Belshe et al. (2007) | 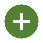 | N.A. | 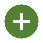 | 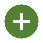 | 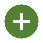 | 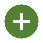 | 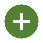 |
| Carr et al. (2011) | 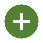 | 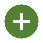 | 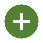 | 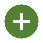 | 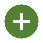 | 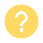 | 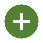 |
| Fleming et al. (2006) | 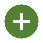 | N.A. | 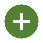 | 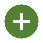 | 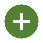 | 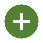 | 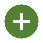 |
| Ilyushina et al. (2015) | 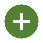 | N.A. | 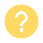 | 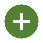 | 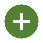 | 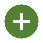 | 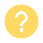 |
| Krishnan et al. (2021) | 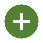 | N.A. | 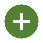 | 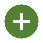 | 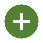 | 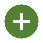 | 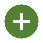 |
| Loeb et al. (2016) | 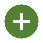 | 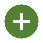 | 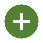 | 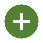 | 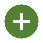 | 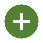 | 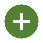 |
| NCT02250274 | 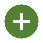 | N.A. | 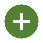 | 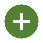 | 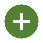 | 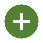 | 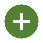 |

# **Supplementary Table 2. Risk of bias assessment of studies included in the safety analysis**

| **Study name** | **Randomization process** | **Timing of identification or recruitment of participants** | **Deviations from intended interventions** | **Missing outcome data** | **Measurement of the outcome** | **Selection of the reported result** | **Overall risk of bias** |
| --- | --- | --- | --- | --- | --- | --- | --- |
| Ashkenazi et al. (2006) | 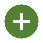 | N.A. | 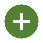 | 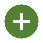 | 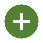 | 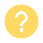 | 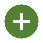 |
| Belshe et al. (2007) | 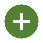 | N.A. | 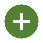 | 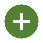 | 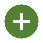 | 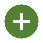 | 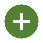 |
| Carr et al. (2011) | 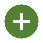 | 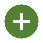 | 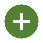 | 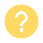 | 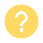 | 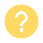 | 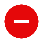 |
| Fleming et al. (2006) | 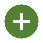 | N.A. | 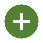 | 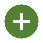 | 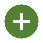 | 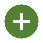 | 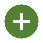 |
| Hoft et al. (2011) | 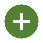 | N.A. | 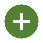 | 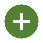 | 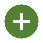 | 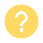 | 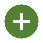 |
| Ilyushina et al. (2015) | 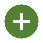 | N.A. | 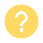 | 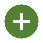 | 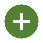 | 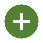 | 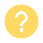 |
| Krishnan et al. (2021) | 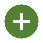 | N.A. | 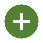 | 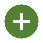 | 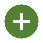 | 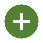 | 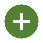 |
| Kwong et al. (2015) | 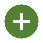 | N.A. | 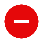 | 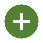 | 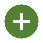 | 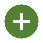 | 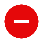 |
| Loeb et al. (2016) | 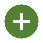 |  |  |  |  |  |  |
| Levin et al. (2008) |  | N.A. |  |  |  |  |  |
| Neuzil et al. (2001) |  |  |  |  |  |  |  |
| Sokolow et al. (2022) |  |  |  |  |  |  |  |
| EU-CTR 2004-000585-13 |  | N.A. |  |  |  |  |  |
| NCT00461981 |  | N.A. |  |  |  |  |  |
| NCT01194297 |  | N.A. |  |  |  |  |  |
| NCT01246999 |  | N.A. |  |  |  |  |  |
| NCT02250274 |  | N.A. |  |  |  |  |  |
| NCT03600428 |  | N.A. |  |  |  |  |  |
| NCT03982069 |  | N.A. |  |  |  |  |  |

# **Supplementary Table 3. Results of the trivalent GRADE assessment**

| **Certainty assessment** | | | | | | | **№ of patients** | | **Effect** | | **Certainty** | |
| --- | --- | --- | --- | --- | --- | --- | --- | --- | --- | --- | --- | --- |
| **№ of studies** | **Study design** | **Risk of bias** | **Inconsistency** | **Indirectness** | **Imprecision** | **Other** | **LAIV** | **IIV** | **Relative (95% CI)** | **Absolute (95% CI)** |  |  |
| **Confirmed influenza cases after vaccination - (assessed with: OR)** | | | | | | | | | | | |  |
| 7 | randomised trials | not serious | serious | not serious | very serious | none | 354/7625 (4.6%) | 581/7531 (7.7%) | **OR 0.77** (0.44 to 1.34) | **17 fewer per 1,000** (from 42 fewer to 24 more) | ⨁◯◯◯ Very low | |
| **Vaccine-related serious adverse events** | | | | | | | | | | | |  |
| 9 | randomised trials | very serious | not serious | not serious | serious | none | 12/8582 (0.1%) | 11/8510 (0.1%) | **OR 1.07** (0.70 to 1.62) | **0 fewer per 1,000** (from 0 fewer to 1 more) | ⨁◯◯◯ Very low | |
| **Serious adverse events (number of affected people)** | | | | | | | | | | | |  |
| 7 | randomised trials | not serious | serious | not serious | serious | none | 107/5908 (1.8%) | 96/5964 (1.6%) | **OR 1.11** (0.96 to 1.28) | **2 more per 1,000** (from 1 fewer to 4 more) | ⨁⨁◯◯ Low | |
| **Serious adverse events (event number)** | | | | | | | | | | | |  |
| 7 | randomised trials | not serious | not serious | serious | not serious | none | 244/7949 (3.1%) | 210/8754 (2.4%) | **OR 1.17** (1.02 to 1.34) | **4 more per 1,000** (from 0 fewer to 8 more) | ⨁⨁⨁◯ Moderate | |
| **Adverse events** | | | | | | | | | | | |  |
| 6 | randomised trials | very serious | very serious | serious | serious | none | 3544/7155 (49.5%) | 3485/6939 (50.2%) | **OR 0.81** (0.36 to 1.83) | **53 fewer per 1,000** (from 236 fewer to 146 more) | ⨁◯◯◯ Very low | |
| **Hospitalization** | | | | | | | | | | | |  |
| 4 | randomised trials | not serious | serious | not serious | extremely serious | none | 153/11896 (1.3%) | 163/12119 (1.3%) | **OR 0.58** (0.04 to 7.86) | **6 fewer per 1,000** (from 13 fewer to 83 more) | ⨁◯◯◯ Very low | |
| **At least subfebrility** | | | | | | | | | | | |  |
| 10 | randomised trials | very serious | very serious | not serious | serious | none | 1063/9452 (11.2%) | 1231/10170 (12.1%) | **OR 0.93** (0.54 to 1.61) | **8 fewer per 1,000** (from 52 fewer to 60 more) | ⨁◯◯◯ Very low | |
| **At least subfebrility under six years of age** | | | | | | | | | | | |  |
| 5 | randomised trials | not serious | very serious^h^ | not serious | very serious | none | 280/5369 (5.2%) | 326/5410 (6.0%) | **OR 1.32** (0.46 to 3.75) | **18 more per 1,000** (from 32 fewer to 134 more) | ⨁◯◯◯ Very low | |
| **At least subfebrility above six years of age** | | | | | | | | | | | |  |
| 3 | randomised trials | serious | not serious | not serious | very serious | none | 55/1582 (3.5%) | 60/1566 (3.8%) | **OR 0.91** (0.58 to 1.43) | **3 fewer per 1,000** (from 16 fewer to 16 more) | ⨁◯◯◯ Very low | |
|  | | | | | | | | | | | |  |
|  | | | | | | | | | | | |  |
|  | | | | | | | | | | | |  |
| **Fever** | | | | | | | | | | | |  |
| 3 | randomised trials | serious | serious | not serious | extremely serious | none | 52/998 (5.2%) | 65/989 (6.6%) | **OR 0.86** (0.05 to 14.77) | **9 fewer per 1,000** (from 62 fewer to 444 more) | ⨁◯◯◯ Very low | |
| **Having a temperature higher than 38,5C** | | | | | | | | | | | |  |
| 3 | randomised trials | not serious | not serious | not serious | very serious | none | 59/1974 (3.0%) | 73/1877 (3.9%) | **OR 0.78** (0.37 to 1.66) | **8 fewer per 1,000** (from 24 fewer to 24 more) | ⨁⨁◯◯ Low | |
| **Sore throat** | | | | | | | | | | | |  |
| 6 | randomised trials | not serious | not serious | not serious | not serious | none | 504/3198 (15.8%) | 458/3194 (14.3%) | **OR 1.14** (0.99 to 1.30) | **17 more per 1,000** (from 1 fewer to 35 more) | ⨁⨁⨁⨁ High | |
| **Significant wheezing** | | | | | | | | | | | |  |
| 3 | randomised trials | not serious | not serious | not serious | not serious | none | 397/6168 (6.4%) | 356/6956 (5.1%) | **OR 1.16** (0.86 to 1.56) | **8 more per 1,000** (from 7 fewer to 26 more) | ⨁⨁⨁⨁ High | |
| **Coughing** | | | | | | | | | | | |  |
| 6 | randomised trials | not serious | not serious | not serious | not serious | none | 1003/2816 (35.6%) | 1024/2826 (36.2%) | **OR 0.95** (0.86 to 1.05) | **12 fewer per 1,000** (from 34 fewer to 11 more) | ⨁⨁⨁⨁ High | |
| **Skin reactions** | | | | | | | | | | | |  |
| 3 | randomised trials | not serious | not serious | serious | extremely serious^k^ | none | 110/4733 (2.3%) | 142/4643 (3.1%) | **OR 1.27** (0.14 to 11.65) | **8 more per 1,000** (from 26 fewer to 238 more) | ⨁◯◯◯ Very low | |
| **Nasal symptoms** | | | | | | | | | | | |  |
| 9 | randomised trials | not serious | serious | not serious | not serious | none | 3347/7525 (44.5%) | 3222/7449 (43.3%) | **OR 1.48** (0.45 to 4.89) | **98 more per 1,000** (from 177 fewer to 356 more) | ⨁⨁⨁◯ Moderate | |
| **Otitis media** | | | | | | | | | | | |  |
| 3 | randomised trials | not serious | serious | not serious | extremely serious^e^ | none | 1446/6344 (22.8%) | 1451/6321 (23.0%) | **OR 1.48** (0.45 to 4.89) | **76 more per 1,000** (from 111 fewer to 363 more) | ⨁◯◯◯ Very low | |
| **Irritability** | | | | | | | | | | | |  |
| 5 | randomised trials | not serious | serious | not serious | very serious | none | 462/4155 (11.1%) | 430/4884 (8.8%) | **OR 1.08** (0.78 to 1.49) | **6 more per 1,000** (from 18 fewer to 38 more) | ⨁◯◯◯ Very low | |
| **Headache** | | | | | | | | | | | |  |
| 6 | randomised trials | not serious | serious | not serious | serious^i^ | none | 651/4203 (15.5%) | 713/4959 (14.4%) | **OR 0.90** (0.62 to 1.31) | **12 fewer per 1,000** (from 49 fewer to 37 more) | ⨁⨁◯◯ Low | |
| **Decreased appetite** | | | | | | | | | | | |  |
| 4 | randomised trials | not serious | very serious | not serious | very serious | none | 583/4567 (12.8%) | 633/5237 (12.1%) | **OR 0.94** (0.66 to 1.35) | **6 fewer per 1,000** (from 38 fewer to 36 more) | ⨁◯◯◯ Very low | |
| **Decreased activity** | | | | | | | | | | | |  |
| 5 | randomised trials | not serious | serious | not serious | serious | none | 543/4133 (13.1%) | 553/4877 (11.3%) | **OR 1.02** (0.59 to 1.76) | **2 more per 1,000** (from 43 fewer to 70 more) | ⨁⨁◯◯ Low | |
| **Vomiting** | | | | | | | | | | | |  |
| 6 | randomised trials | not serious | serious | not serious | not serious | none | 354/5731 (6.2%) | 394/6421 (6.1%) | **OR 1.09** (0.78 to 1.53) | **5 more per 1,000** (from 13 fewer to 30 more) | ⨁⨁⨁◯ Moderate | |
| **Upper respiratory tract infections** | | | | | | | | | | | |  |
| 4 | randomised trials | serious | serious | not serious | extremely serious | none | 296/5368 (5.5%) | 278/5358 (5.2%) | **OR 1.64** (0.27 to 10.15) | **30 more per 1,000** (from 37 fewer to 305 more) | ⨁◯◯◯ Very low | |
| **Nasopharyngitis** | | | | | | | | | | | |  |
| 4 | randomised trials | not serious | very serious | not serious | very serious | none | 151/5466 (2.8%) | 132/5458 (2.4%) | **OR 1.22** (0.53 to 2.79) | **5 more per 1,000** (from 11 fewer to 40 more) | ⨁◯◯◯ Very low | |
| **Muscle or body ache** | | | | | | | | | | | |  |
| 5 | randomised trials | not serious | serious | not serious | very serious | none | 226/2210 (10.2%) | 278/2151 (12.9%) | **OR 0.56** (0.24 to 1.32) | **53 fewer per 1,000** (from 95 fewer to 35 more) | ⨁◯◯◯ Very low | |
| **Ear and eye reactions** | | | | | | | | | | | |  |
| 4 | randomised trials | not serious | not serious | serious | very serious | none | 197/4794 (4.1%) | 206/4707 (4.4%) | **OR 1.06** (0.47 to 2.40) | **3 more per 1,000** (from 23 fewer to 55 more) | ⨁◯◯◯ Very low | |
| **Diarrhoea** | | | | | | | | | | | |  |
| 4 | randomised trials | not serious | not serious | not serious | not serious | none | 617/6689 (9.2%) | 684/7386 (9.3%) | **OR 0.98** (0.83 to 1.16) | **2 fewer per 1,000** (from 15 fewer to 13 more) | ⨁⨁⨁⨁ High | |
| **Chills** | | | | | | | | | | | |  |
| 4 | randomised trials | not serious | serious | not serious | serious | none | 207/2208 (9.4%) | 213/2143 (9.9%) | **OR 0.74** (0.26 to 2.11) | **24 fewer per 1,000** (from 71 fewer to 89 more) | ⨁⨁◯◯ Low | |
| **Wheezing** | | | | | | | | | | | |  |
| 5 | randomised trials | not serious | serious | not serious | very serious | none | 839/8331 (10.1%) | 857/9077 (9.4%) | **OR 0.98** (0.73 to 1.33) | **2 fewer per 1,000** (from 24 fewer to 27 more) | ⨁◯◯◯ Very low | |
| **Adverse events (number of events)** | | | | | | | | | | | |  |
| 3 | randomised trials | serious | serious^m^ | not serious | serious | none | 2788/4711 (59.2%) | 2857/4620 (61.8%) | **OR 0.51** (0.05 to 5.09) | **166 fewer per 1,000** (from 543 fewer to 273 more) | ⨁◯◯◯ Very low | |
| **Adverse events (number of affected people)** | | | | | | | | | | | |  |
| 3 | randomised trials | serious | not serious | not serious | not serious | none | 756/2444 (30.9%) | 628/2319 (27.1%) | **OR 1.26** (1.14 to 1.40) | **48 more per 1,000** (from 27 more to 71 more) | ⨁⨁⨁◯ Moderate | |
| **Nasal symptoms – large, multi-center studies** | | | | | | | | | | | |  |
| 6 | randomised trials | serious | serious | not serious | serious | none | 167/1193 (14.0%) | 149/1155 (12.9%) | **OR 1.24** (0.89 to 1.71) | **26 more per 1,000** (from 13 fewer to 73 more) | ⨁◯◯◯ Very low | |
| **Nasal symptoms – smaller studies** | | | | | | | | | | | |  |
| 3 | randomised trials | not serious | not serious | not serious | not serious | none | 3830/6332 (60.5%) | 3073/6294 (48.8%) | **OR 1.64** (1.33 to 2.02) | **122 more per 1,000** (from 71 more to 170 more) | ⨁⨁⨁⨁ High | |
| **Vaccine-related serious adverse events under nine years of age** | | | | | | | | | | | |  |
| 5 | randomised trials | not serious | not serious | not serious | serious | none | 8/6358 (0.1%) | 9/6336 (0.1%) | **OR 0.93** (0.53 to 1.63) | **0 fewer per 1,000** (from 1 fewer to 1 more) | ⨁⨁⨁◯ Moderate | |
| **Confirmed influenza cases after vaccination – large, multi-center studies** | | | | | | | | | | | |  |
| 3 | randomised trials | not serious | not serious | not serious | not serious | none | 232/6077 (3.8%) | 471/6077 (7.8%) | **OR 0.50** (0.28 to 0.88) | **37 fewer per 1,000** (from 55 fewer to 9 fewer) | ⨁⨁⨁⨁ High | |
| **Confirmed influenza cases after vaccination – smaller studies** | | | | | | | | | | | |  |
| 4 | randomised trials | not serious | serious | not serious | not serious | none | 167/1548 (10.8%) | 110/1454 (7.6%) | **OR 1.49** (1.18 to 1.89) | **33 more per 1,000** (from 12 more to 58 more) | ⨁⨁⨁◯ Moderate | |

**CI:** confidence interval; **OR:** odds ratio

# **Supplementary Table 4. Results of the quadrivalent GRADE assessment**

| **Certainty assessment** | | | | | | | **№ of patients** | | **Effect** | | **Certainty** | |
| --- | --- | --- | --- | --- | --- | --- | --- | --- | --- | --- | --- | --- |
| **№ of studies** | **Study design** | **Risk of bias** | **Inconsistency** | **Indirectness** | **Imprecision** | **Other** | **LAIV** | **IIV** | **Relative (95% CI)** | **Absolute (95% CI)** |  |  |
| **Related serious adverse events (same for Serious adverse events by the number of affected people and by event number)** | | | | | | | | | | | |  |

| 4 | randomised trials | serious | not serious | not serious | extremely serious | none | 0/395 (0.0%) | 0/346 (0.0%) | **OR 0.92** (0.46 to 1.87) | **0 fewer per 1,000** (from 0 fewer to 0 fewer) | ⨁◯◯◯ Very low |
| --- | --- | --- | --- | --- | --- | --- | --- | --- | --- | --- | --- |

**CI:** confidence interval; **OR:** odds ratio

# **Supplementary Table 5 . Definitions of serious adverse events reported in the included studies**

| Study | Health status of children | Period | Definitions |
| --- | --- | --- | --- |
| Ashkenazi et al. (2006) | history of recurrent respiratory tract infections | 0-end of study | Serious AEs (SAEs), including hospitalizations, were monitored from enrollment through completion of the study. |
| Belshe et al. (2007) | healthy+wheezer 6% | 0-end of study | Serious adverse events (defined as events that were lifethreatening or that resulted in death, hospitalization or prolonged hospitalization, significant disability or incapacity, or another important medical event requiring intervention to prevent one of these outcomes) were collected from the day of dose 1 until the end. |
| Carr et al. (2011) | cancer | 0-180 days | Serious adverse events were collected for 180 days. |
| Krishnan et al. (2021) | around 50% malnourished | 0-end of study (2 years) | Surveillance for serious adverse events (SAEs) defined as events resulting in death, hospitalization, immediately life-threaten ing conditions, or persistent or significant disabilities continued throughout the 2-year study period. |
| NCT00461981 |  | NI | An adverse event that results in death, is life-threatening, requires inpatient hospitalization or extends a current hospital stay, results in an ongoing or significant incapacity or interferes substantially with normal life functions, or causes a congenital anomaly or birth defect. Medical events that do not result in death, are not life-threatening, or do not require hospitalization may be considered serious adverse events if they put the participant in danger or require medical or surgical intervention to prevent one of the results listed above. |
| NCT01194297 | premature, very low birth weight and former full-term infants | 0-42 days |  |
| NCT01246999 | healthy | NI |  |
| NCT02250274 | healthy | NI |  |
| NCT03600428 | asthma | 0-42 days |  |
| NCT03982069 | healthy | 5 weeks |  |

# **Supplementary Table 6. Definitions of adverse- and reactogenicity events reported in the included studies**

| Study | Health status of children | Period | Definitions |
| --- | --- | --- | --- |
| Ashkenazi et al. (2006) | history of recurrent respiratory tract infections | Day 0-10 | Any clinically significant untoward, undesired, or unex- pected event, including those that required prescription or nonprescription medication within 11 days postvaccination (days 0–10). |
|  |  | Day 0-27 | An event requiring an unscheduled healthcare provider visit or consultation within 28 days of vaccination. |
|  |  | Day 0-end | An event resulting in study termination or a clinically significant event at any point during the study period. |
| Belshe et al. (2007) | healthy+wheezer 6% | Day 0-41 | Parents or guardians recorded local reactions, daily temperatures (oral, axillary, or rectal), systemic adverse events, and concomitant medications on worksheets. |
| Carr et al. (2011) | cancer | Day 0-27 | National Cancer Institute Common Toxicity Criteria and relationship to vaccination:  Fever, rhinorrhea/nasal congestion, sore throat, cough, vomiting, headache, muscle aches, pain, chills, tiredness, and irritability in a diary card for 28 consecutive days after each vaccination. For subjects receiving TIV, the presence or absence of redness, swelling, and/or pain around the injection site was also recorded. |
| Fleming et al. (2006) | asthma | 0-14 | Any clinically significant event (following administration of the vaccine dose), including but not limited to the following events: (1) those that required prescription or nonprescription medication within 15 days postvaccination (days 0–14) |
|  |  | 0-27 | (2) those that required an unscheduled healthcare provider visit or consultation within 28 days of vaccination |
|  |  | 0-end | (3) those that resulted in study termination or (4) any other clinically significant event occurring at any point during the study period. |
|  |  | 0-15 | Reactogenicity events—predefined events that could occur after vaccine administration—were recorded for 15 consecutive days after study vaccination. Events to be re- corded were fever (oral temperature 38°C), runny nose/ nasal congestion, sore throat, cough, wheeze, vomiting, change in activity level, appetite change, irritability, abdom- inal pain/stomachache, headache, chills, and muscle aches. For subjects receiving TIV, the presence or absence of red- ness, swelling, and/or pain around the injection site was also recorded. Reactogenicity events that met the criteria for an AE or SAE as described above were also recorded as such. |
| Hoft et al. (2011) | healthy | 0-14 | After vaccination, subjects were observed for 30 minutes for adverse reactions. Parents and guardians were given memory aids to record adverse events for 2 weeks after vaccination. The memory aid included scoring of solicited systemic and local reactions and prompted the parent to call in case of a severe reaction. |
| Ilyushina et al. (2015) | healthy | NI | Reactogenicity events are defined as pain, tenderness, fatigue, nose bleed, red eyes after italicized dose. |
| Krishnan et al. (2021) | around 50% malnourished | 0-42 | All children were observed for 30 minutes after each vaccination and visited at home on  days 1, 3, 7, 14, 21, 28, and 42 to monitor adverse events. |
| Levin et al. (2008) | healthy | 3-21 | Subjects or their caretakers had information collected about adverse events on days 3, 7, 14, and 21 post-vaccination by telephone if they were in Arm B, and by telephone (days 7 and 21) and during scheduled study visits (days 3 and 14) if they were in Arm A. Subjects in both Arms were seen in clinic on day 28 post-vaccination. |
| Loeb et al. (2016) | healthy | Day 0-4 | NI |
|  |  | Day 0-end | Passive surveillance for adverse reactions to the  vaccine was implemented throughout the study period. |
| Neuzil et al. (2001) | healthy | Day 0-4 | All subjects were asked to complete a vaccine reaction form to indicate any systemic or local symptoms during the 4 days after immunization. |
| Sokolow et al. (2022) | asthma | Day 0-14 | Local and systemic reactogenicity events, asthma symptoms, unscheduled albuterol use, PEFR measurements, and medical utilization were captured daily through 14 days after vaccination. |
| EU-CTR 2004-000585-13 | healthy | Day 0-180 | An adverse event (AE) was any untoward medical occurrence in a participant who received study drug without regard to possibility of causal relationship. Treatment-emergent are events between administration of study drug and up to Day 180 that were absent before treatment or that worsened relative to pretreatment state. |
| NCT00461981 | healthy | Day 0-41 | An adverse event that is not a serious adverse event, meaning that it does not result in death, is not life-threatening, does not require inpatient hospitalization or extend a current hospital stay, does not result in an ongoing or significant incapacity or interfere substantially with normal life functions, and does not cause a congenital anomaly or birth defect; it also does not put the participant in danger and does not require medical or surgical intervention to prevent one of the results listed above. |
| NCT01194297 | premature, very low birth weight and former full-term infants | NI |  |
| NCT03982069 | healthy | 5 weeks |  |

# **Supplementary Figure1. Leave-one-out Sensitivity Analysis regarding the efficacy of influenza vaccines**

# **Supplementary Figure2. Detailed forest plots of the safety analyses**

# **We compared the incidence of different safety features after influenza vaccination presented in odds ratios (results of trivalent versus trivalent-, and quadrivalent versus quadrivalent vaccine studies are presented separately)**

*LAIV: live-attenuated influenza vaccine*

*IIV: inactivated intramuscular influenza vaccine*

## **All-cause mortality**

This figure summarizes the odds ratios comparing the incidence of all-cause mortality after vaccination between IIV and LAIV stratified by subgroups based on vaccine valency (Trivalent or Quadrivalent).

## **Serious adverse events**

### Serious adverse events (event number)

This figure summarizes the odds ratios comparing the incidence of serious adverse events (reported as event number, rather than as the number of people reporting it) after vaccination between IIV and LAIV stratified by subgroups based on vaccine valency (Trivalent or Quadrivalent).

1. Serious adverse events (number of affected persons)

This figure summarizes the odds ratios comparing the incidence of serious adverse events (reported as the number of people reporting them, rather than the cumulative event number) after vaccination between IIV and LAIV stratified by subgroups based on vaccine valency (Trivalent or Quadrivalent).

### Vaccine-related serious adverse events

### This figure summarizes the odds ratios comparing the incidence of those serious adverse events, which’s occurrence can be directly related to influenza vaccination (study conductor’s opinion) after vaccination between IIV and LAIV stratified by subgroups based on vaccine valency (Trivalent or Quadrivalent

### Vaccine-related serious adverse events under nine years (trivalent vaccines)

This figure summarizes the odds ratios comparing the incidence of those serious adverse events, which’s occurrence can be directly related to influenza vaccination (study conductor’s opinion) for children younger than nine years old.

## **Hospitalization**

This figure summarizes the odds ratios comparing the incidence of hospitalization after vaccination between trivalent IIV and LAIV.

## **Adverse events**

## This figure summarizes the odds ratios comparing the incidence of adverse events after vaccination between trivalent IIV and LAIV stratified by subgroups based on adverse event reporting (“Based on the number of affected people”: events were reported based on the number of people who experienced adverse events throughout a period of time regardless of the number of episodes; “Based on event number”: event number was reported regardless of the number of reporting people).

## **Asthma**

**This** figure summarizes the odds ratios comparing the incidence of asthma exacerbation after vaccination between IIV and LAIV stratified by subgroups based on vaccine valency (trivalent or quadrivalent**).**

## **At least subfebrility**

### Main analysis

This figure summarizes the odds ratios comparing the incidence of temperature elevations of at least subfebrile after vaccination between trivalent IIV and LAIV.

### Subgroup analysis based on age (6 years)

This figure summarizes the odds ratios comparing the incidence of asthma exacerbation after vaccination between trivalent IIV and LAIV stratified by subgroups based on age (under or above six years)

## **Chills**

This figure summarizes the odds ratios comparing the incidence of chills after vaccination between trivalent IIV and LAIV.

## **Coughing**

This figure summarizes the odds ratios comparing the incidence of coughing after vaccination between IIV and LAIV stratified by subgroups based on vaccine valency (trivalent or quadrivalent).

## **Decreased activity**

This figure summarizes the odds ratios comparing the incidence of decreased activity after vaccination between trivalent IIV and LAIV.

## **Decreased appetite**

This figure summarizes the odds ratios comparing the incidence of decreased appetite after vaccination between trivalent IIV and LAIV.

## **Diarrhea**

This figure summarizes the odds ratios comparing the incidence of diarrhea after vaccination between trivalent IIV and LAIV.

## **Ear or eye reactions**

This figure summarizes the odds ratios comparing the incidence of ear or eye reactions after vaccination between trivalent IIV and LAIV.

## **Fever**

This figure summarizes the odds ratios comparing the incidence of fever after vaccination between trivalent IIV and LAIV.

## **Having a temperature higher than 38.5C**

This figure summarizes the odds ratios comparing the incidence having a temperature higher than 38.5C after vaccination between trivalent IIV and LAIV.

## **Headache**

This figure summarizes the odds ratios comparing the incidence of headache after vaccination between trivalent IIV and LAIV.

## **Irritability**

This figure summarizes the odds ratios comparing the incidence of irritability after vaccination between trivalent IIV and LAIV.

## **Muscle- or body ache**

This figure summarizes the odds ratios comparing the incidence of muscle- or body ache after vaccination between trivalent IIV and LAIV.

## **Nasal symptoms**

This figure summarizes the odds ratios comparing the incidence of coughing after vaccination between trivalent IIV and LAIV stratified by study size (more ir less than a 100 centers).

## **Nasopharyngitis**

This figure summarizes the odds ratios comparing the incidence of nasopharyngitis after vaccination between trivalent IIV and LAIV.

## **Otitis media**

This figure summarizes the odds ratios comparing the incidence of otitis media after vaccination between trivalent IIV and LAIV.

## **Skin reactions**

This figure summarizes the odds ratios comparing the incidence of skin reactions after vaccination between trivalent IIV and LAIV.

## **Sore throat**

This figure summarizes the odds ratios comparing the incidence of sore throat after vaccination between trivalent IIV and LAIV.

## **Upper respiratory tract infections**

This figure summarizes the odds ratios comparing the incidence of upper respiratory tract infections after vaccination between trivalent IIV and LAIV.

## **Vomiting**

This figure summarizes the odds ratios comparing the incidence of vomiting after vaccination between trivalent IIV and LAIV.

## **Wheezing**

### Main analysis

This figure summarizes the odds ratios comparing the incidence of wheezing after vaccination between IIV and LAIV, stratified by subgroups based on vaccine valency (Trivalent or Quadrivalent)

### Significant wheezing

This figure summarizes the odds ratios comparing the incidence of wheezing of significant level after vaccination between IIV and LAIV, stratified by subgroups based on vaccine valency (Trivalent or Quadrivalent).

# **Publication bias, funnel plots, Egger’s tests**

## **Supplementary Figure 3. Funnel plot of the nasal symptoms analysis**

##

## **Supplementary Figure 4. Funnel plot of the vaccine-related serious adverse events analysis**

## **Supplementary Figure 5. Funnel plot and Eggers’ test of the “at least subfebrility” analysis**

Review: SSI

Linear regression test of funnel plot asymmetry

Test result: t = -0.13, df = 8, p-value = 0.9006

Sample estimates:

bias se.bias intercept se.intercept

-0.1879 1.4579 0.0636 0.2251

Details:

- multiplicative residual heterogeneity variance (tau^2 = 10.2427)

- predictor: standard error

- weight: inverse variance

- reference: Egger et al. (1997), BMJ

## **Supplementary Figure 6. Funnel plot of the “at least subfebrility ” subgroup analysis (younger or older than six years)**
